# Physicochemical Characteristics of Residual Carbon and Inorganic Minerals in Coal Gasification Fine Slag

**DOI:** 10.3390/molecules29163956

**Published:** 2024-08-21

**Authors:** Le Li, Jing Liu, Xiangyang Li, Zeyu Peng, Chun Han, Wenhao Lian, Bin Xue, Chenmin Gao, Qian Zhang, Wei Huang

**Affiliations:** 1 State Key Laboratory of Clean and Efficient Coal Utilization, Taiyuan University of Technology, Taiyuan 030024, China; lixiangyang0913@link.tyut.edu.cn (X.L.); david13834557950@163.com (Z.P.); 15552201638@163.com (C.H.); xuebin0716@link.tyut.edu.cn (B.X.); 13593199081@163.com (C.G.); zhangqian01@tyut.edu.cn (Q.Z.); 2 Department of Chemistry and Chemical Engineering, Shanxi Polytechnic College, Taiyuan 030032, China; liujing951123@163.com; 3 School of Chemical Engineering and Technology, North University of China, Taiyuan 030024, China; wlian@nuc.edu.cn

**Keywords:** coal gasification fine slag, residual carbon, inorganic minerals, physicochemical characteristics

## Abstract

Investigating the physicochemical properties and embedding forms of residual carbon (RC) and slag particles (SPs) in coal gasification fine slag (FS) is the basis for achieving its separation and utilization. An in-depth understanding of their compositional characteristics allows for targeted treatment and utilization programs for different components. In this work, the physicochemical properties and embedding forms of RC and SPs in FS were systematically investigated. An innovative calculation method is proposed to determine the mass fraction of dispersed carbon particles, dispersed mineral-rich particles, and carbon–ash combined particles by using a high-temperature heating stage coupled with an optical microscope. The unburned RC with a rough, loose surface and a well-developed pore structure acted as a framework in which the smaller spherical SPs with a smooth surface were embedded. In addition, the sieving pretreatment process facilitated the enrichment of the RC. Moreover, the RC content showed significant dependencies according to the FS particle size. For FS with a particle size of 0.075–0.150 mm, the mass proportions of dispersed carbon, ash particles, and the carbon–ash combination were 15.19%, 38.72%, and 46.09%, respectively. These findings provide basic data and reliable technical support for the subsequent carbon and ash separation process and the comprehensive utilization of coal gasification slag.

## 1. Introduction

Coal gasification, a pivotal technology for the clean and efficient utilization of coal, is widely employed in the modern coal chemical industry [1,2,3]. However, as coal is not entirely gasified during this process, the residual carbon (RC) combines with minerals and other impurities to form a solid residue, i.e., coal gasification slag (CGS) [4], whose annual emission exceeds 50 million tons. Currently, CGS is mainly disposed of by stacking and landfilling, which lead to severe pollution of the environment and the wastage of land resources, hindering the sustainable development of coal chemical enterprises. Therefore, developing high-value and large-scale resource utilization methods of CGS is imperative.

CGS can typically be divided into coarse slag (CS) [5] and fine slag (FS) [6] based on the generation method and physical properties. CS, comprising 60% to 80% of the total gasification slag, can be directly used as the raw material for construction materials due to its low unburned carbon content (<10%) [7]. Nevertheless, FS, accounting for 20% to 40% of the total gasification slag, exists in the form of irregularly shaped particles with smaller sizes (0–1 mm) and has a higher residual carbon content (>40%) [8], making it unsuitable for direct utilization due to severe environmental pollution. High-level RC can be used to make high-value products [9,10,11] (e.g., catalysts, porous materials, and plastic fillers) with resource and environmental benefits. Therefore, the development of cleaner and high-efficiency methods for separating and recycling RC from FS has become a top priority for the high-value utilization of FS.

The key to separating RC and slag particles (SPs) lies in understanding the physicochemical properties (such as shape, density, and surface properties) and clarifying the complex embedding forms of RC and SPs. So far, most studies [12,13] have primarily focused on the physicochemical properties of FS, with little attention given to the embedding forms of RC and SPs within the FS. Han et al. [14] found that different gasification techniques led to differences in surface properties of FS with different particle sizes. The gasification process affected the surface functional groups of FS, with lower types and numbers of oxygen-containing functional groups when the pressure and temperature were higher. Li et al. [12] discovered that RC might originate from partially pyrolyzed or incompletely gasified carbon, characterized by a notably high pore-specific surface area and a significant average pore size. Miao et al. [15] discovered that RC existed in three forms: discrete, embedded in SPs, and crosslinked SPs. Furthermore, Niu et al. [16] found that residual carbon associated with and bonded to SPs was also present in FS. The above literature results reveal that the embedded forms as well as the properties of RC and SPs were closely related to the process of coal gasification and the particle size of the FS. Therefore, determining the appropriate separation technology based on specific embedded forms is crucial for improving the RC enrichment efficiency.

At present, the primary process for separating RC and SPs from FS includes gravity separation [17,18,19] and flotation [20,21,22]. Gravity separation exploits the density disparity between RC and SPs, enabling their separation based on their distinct displacement in a gravitational field. Although the method is both simple and conducive to large-scale production, it is not applicable to materials with minimal differences in particle size and density, as they have similar terminal velocities, increasing the possibility of particle misalignment [19]. Flotation achieves effective particle separation by taking advantage of the differences in hydrophilicity and hydrophobicity between raw material components. Compared with traditional flotation, the use of ultrasonic pretreatment of the pulp enhanced the selectivity of the collector [21]. The enrichment effect of small RC particles (0.00–0.15 mm) was notably improved by augmenting the fragmentation effect of FS. However, problems with the flotation method still exist, such as the high consumption of chemicals and the poor sorting effect on small-size particles [23].

Improving the carbon extraction efficiency of FS in the above separation process requires an in-depth investigation of the physicochemical properties of FS particles. Hence, in this study, the physicochemical properties, including RC content, particle size distribution, surface characteristics, pore structure, ash composition, crystal mineral content, and embedding forms, of RC and SPs in FS were investigated by using a particle size analyzer, SEM, the N_2_ adsorption technique, XRD, and a high-temperature heating stage coupled with an optical microscope. An innovative calculation method is proposed to determine the mass fraction of dispersed carbon particles, dispersed mineral-rich particles, and combined carbon and ash particles. These findings are important supplements to understanding the structures of FS, RC, and SPs, providing reliable technical support for effective carbon–ash separation and the subsequent comprehensive utilization of gasification slag.

## 2. Results and Discussion

### 2.1. Physicochemical Characteristics

#### 2.1.1. Proximate and Ultimate Analysis

The industrial analyses of FS and RC samples were determined in muffle furnaces (DTXL-2000, Taian Yucheng Mining Equipment Co., Ltd., Taian, China) and ovens (101-2EBS, Beijing Hifuta Technology Co., Ltd., Beijing, China) according to the Chinese national standard GB/T 212-2008 [24]. The elemental analyses of FS and RC samples were determined in an automatic sulfur-measuring instrument (ZCS-8, Hebi Huawei Keli Coal Quality Instrument Co., Ltd., Hebi, China), a carbon- and hydrogen-measuring instrument (CTCH500, Xuzhou Terry Instrument Co., Ltd., Xuzhou, China), and a Kjeldahl nitrogen analyzer (K1100, Shanghai Haineng Experimental Instrument Technology Co., Ltd., Shanghai, China) according to the Chinese national standard GB/T 476-2008 [25]. Their basic properties are described in Table 1. The H content, N content, and volatile matter in the FS were significantly reduced, indicating that the organic matter in the coal had been basically decomposed in the gasifier [26]. Nevertheless, since the gasification process was an under-oxygenated process, the fixed carbon content of FS exceeded 30%, indicating the presence of significant combustible components [27]. The ash content was 63.03%, indicating the presence of a large number of inorganic mineral components [28]. In addition, the fixed carbon content of RC was elevated to 92.28% and the ash content was reduced to 3.22%, indicating that the acid treatment process had maximized the removal of inorganic elements from FS [29].

#### 2.1.2. Ash Composition Analysis

The chemical compositions of FS, SPs, and RC were studied by using a sequential X-ray fluorescence spectrometer (HeLeeX E3-P, Thermo Fisher Scientific, Waltham, MA, USA), and the major elemental oxides are shown in Table 2. The main components of FS and SPs were SiO_2_, Al_2_O_3_, Fe_2_O_3_, and CaO, with basically the same content. The higher silica and aluminum content indicated that its mineral composition contained a large amount of kaolin, quartz, and other minerals [30], resulting in a highly hydrophilic FS [27]. The higher iron and calcium content came from the addition of co-solvents during the gasification process and minerals in the gasified coal [31]. Since the total mass fraction of silicon, aluminum, and iron in the SPs was more than 65%, the FS was expected to be used as a building material after the carbon and ash separation treatment [32]. In addition, the true densities of FS, SPs, and RC were measured to be 2.35 g/cm^3^, 1.94 g/cm^3^, and 2.72 g/cm^3^, respectively, measured on a true density meter (AccuPyc II 1340, Micromeritics Instrument Corp., Norcross, GA, USA) using the gas displacement method.

#### 2.1.3. RC Content Analysis

During gasification, the particle size was affected by mineral fragmentation and coalescence and char combustion, fragmentation, and expansion [33], resulting in different sizes of particles exhibiting different characteristics [31]. The yield and loss on ignition (LOI) of the FS with different particle sizes are shown in Table 3. The yield and LOI of the FS showed significant dependencies according to the particle size. This was related both to the homogeneity of the gas–solid phase contact during the reaction and to the size of the feed coal particles [34]. On the whole, with the increase in the FS particle size, the LOI showed a trend of increasing and then decreasing, suggesting that the sieving pretreatment process facilitated the enrichment of RC, a finding that is consistent with those reported in other works [28,35,36]. Specifically, in this work, the largest fraction (>0.500 mm), whose formation is attributed to the aggregation of molten coal and ash particles during the gasification process [37], has a very low yield, although it contains almost 44% RC. The medium-sized (0.150−0.500 mm) fraction possessed the most yield and more than 60% RC. The smaller-sized (0.075−0.150 mm) fraction contained almost 40% RC. However, the smallest (<0.075 mm) fraction contained the least RC, which might be due to the complete reaction of some coal particles in the gasifier [38]. Hence, the LOI content of FS with a particle fraction of >0.075 mm was significantly higher than that of the <0.075 mm fraction, showing that the RC easily gathers in relatively larger FS fractions.

#### 2.1.4. Particle Size Distributions

**Figure 1:**
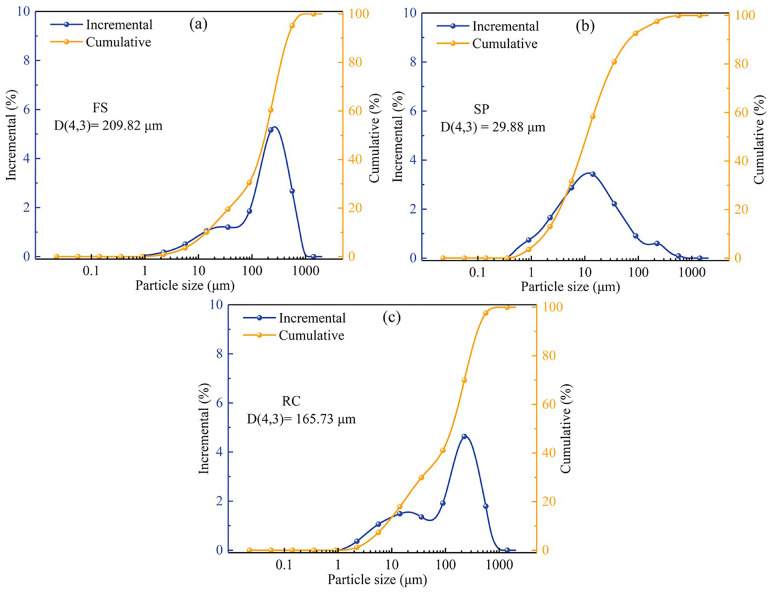
Particle size distribution of (**a**) FS, (**b**) SPs, and (**c**) RC particles.

The particle size distributions of FS, SPs, and RC are shown in Figure 1. The particle size distribution of FS showed a bimodal distribution, with most of the particles concentrated in the range of 100–500 μm, which is different to the trimodal size distribution of FS reported by Gao et al. [39]. The reason for this may be the different types of coal used for the gasification process [34]. The average particle size of the FS was 209.82 μm, as shown in Figure 1a. The particle size distribution of SPs showed a normal distribution, with an average particle size of 29.88 μm, as shown in Figure 1b. Specifically, the particle size distribution of the RC was similar to that of the FS, with an average particle size of 165.73 μm, as shown in Figure 1c. Moreover, no significant variation in the particle size distribution of the RC and FS was observed after acid washing with an ash content of 63.03% in the FS (see Table 1), indicating that most of the small-sized mineral particles were located in the pore structure of large-sized RC particles, which acted as a framework in the FS. Guo et al. [38] drew a similar conclusion that small-sized spherical mineral particles tended to adhere to the pores of large-sized carbon particles, resulting in an increase in pore roughness with an increase in the FS particle size. In addition, the above results also reveal that the acid treatment could remove most of the mineral particles from the macro-, meso-, and micropores in the FS samples without destroying the well-developed pore structure of the RC.

#### 2.1.5. Pore Structure

**Figure 2:**
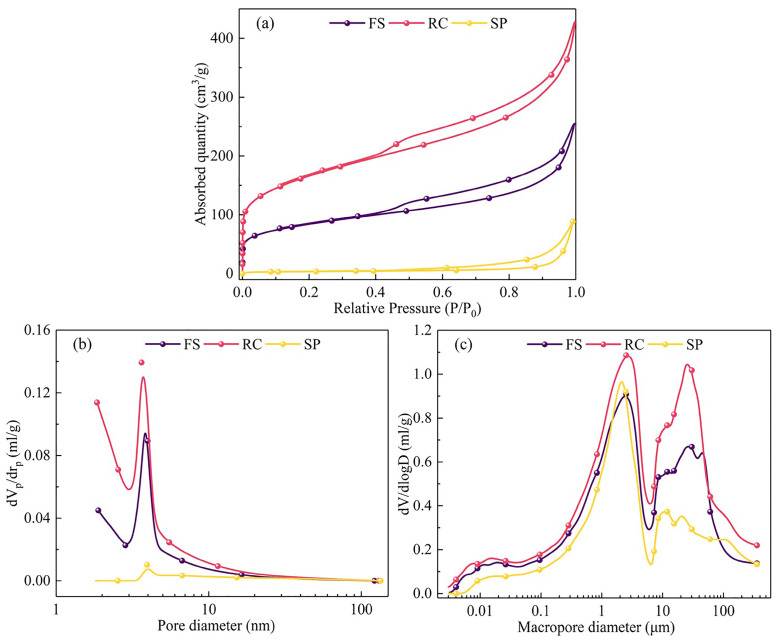
(**a**) Adsorption/desorption isotherms, (**b**) microporous size distribution, and (**c**) macroporous size distribution of the FS, RC, and SPs.

Figure 2 presents the adsorption/desorption isotherms and the pore size distribution of the FS, RC, and SPs. Table 4 lists the surface area, pore volume, and pore size of the FS, RC, and SPs, respectively. As shown in Figure 2a, the nitrogen adsorption/desorption of the FS, RC, and SPs were classified as Type II according to the IUPAC classification [37], which is consistent with reports in the literature [40]. For the FS and RC, significant H4-type hysteresis loops were observed, indicating the presence of narrow slit-like pore structures in both the FS and RC. Similar conclusions were drawn in the work of Liu et al. [28]. At the low-pressure stage (0.01 < *P*/*P*_0_ < 0.1), the nitrogen adsorption increased rapidly, indicating that more microporous (<2 nm) structures existed. Moreover, the microporous distributions of RC were more developed than that of the FS (Figure 2b). However, few micropores were found in the SPs. At the medium-pressure stage (0.1 < *P*/*P*_0_ < 0.8), the nitrogen adsorption increased gradually as the relative pressure increased, indicating the presence of mesoporous (2–50 nm) structures, which were distributed around 4 nm (Figure 2b). At the high-pressure stage (0.8 < *P*/*P*_0_ < 1.0), the nitrogen adsorption increased rapidly, indicating the presence of a macroporous (>50 nm) structure. Moreover, the RC showed higher nitrogen adsorption than the FS (Figure 2a), demonstrating a complete and continuous pore structure that was more developed in the RC than in the FS [38]. The developed pore structure in the RC was conducive to improving the diffusion rate of oxygen and enhancing the combustion reactivity of the FS [41]. In addition, the RC had the largest specific surface area and pore volume (Table 4), indicating that the development of pore structures led to the enlargement of the average pore size. Moreover, as shown in Figure 2c, the macropores in the RC were mainly distributed around 2 μm and 30 μm and the macropores in the SPs were mainly distributed around 2 μm and 10 μm. For the SPs, due to the low nitrogen adsorption, the pores were the least developed. In addition, the SPs had the smallest specific surface area and pore volume (Table 4), indicating that the collapse of pore structures during the gasification reaction caused a decrease in the specific surface area.

#### 2.1.6. Surface Characteristics

**Figure 3:**
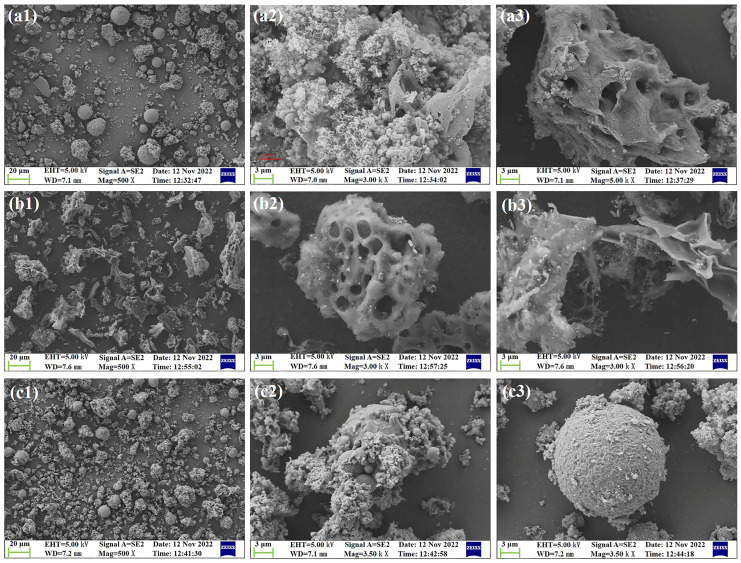
SEM images of the (**a**) FS, (**b**) RC, and (**c**) SPs (the resolution in 1 is 20 μm, while the resolution in 2 and 3 is 3 μm).

The SEM images of the FS, SPs, and RC are shown in Figure 3. The FS was mainly composed of spherical particles and porous particles, in which the spherical particles with a smooth surface were formed by the rapid cooling of basic components in the slag at high temperatures, such as SiO_2_, Al_2_O_3_, and CaO [42], whereas the porous particles in the RC had a rough, loose surface and a well-developed pore structure [34]. Moreover, some of the SPs with a smaller average particle size (29.88 μm, Figure 1b) were embedded in the pore structure of the RC particles, as shown in Figure 3(a2,a3), resulting in the surface roughness of the FS increasing with an increase in the particle size. A similar phenomenon was observed by Guo et al. [38]. The RC was mainly composed of flocculent and porous block structure particles with essentially no spherical particles attached, as shown in Figure 3b. In Figure 3c, two morphologies of SP particles can be observed, i.e., spherical particles and aggregates. Among them, the surface of the spherical particles was smooth without any pore structure (Figure 3(c3)), while the surface of the aggregates was rough (Figure 3(c2)), showing that the inorganic matter in the FS tends to form spherical particles due to surface tension [34] and confirming the results shown in Figure 2c.

#### 2.1.7. Mineral Constituents and Functional Groups

**Figure 4:**
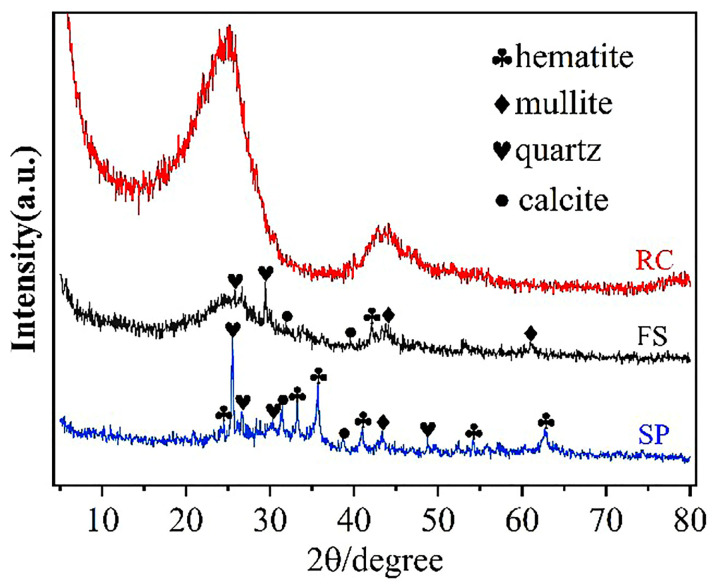
XRD pattern of the FS, RC, and SPs.

Figure 4 shows the XRD pattern of the FS, RC, and SPs. The diffraction peaks of hematite, mullite, quartz, and calcite can be observed in the FS and SPs, and there were no other obvious diffraction peaks, indicating that the hematite, mullite, quartz, and calcite were the main components in the FS and SPs, leading to higher silica and aluminum content (Table 5). The above results confirm that there are more crystalline minerals in FS produced by a GSP entrained-flow pulverized coal gasifier [30]. Moreover, the presence of hematite and the higher iron content in the FS and SPs provide theoretical support for the process of iron extraction from FS [35]. In the XRD diagram of the RC, there are two peaks around 26 degrees and 44 degrees, which correspond to the (002) and (100) crystal planes of the graphite structure, respectively, indicating that the RC had been partially graphitized after the high-temperature treatment during the generation of gasification slag.

**Figure 5:**
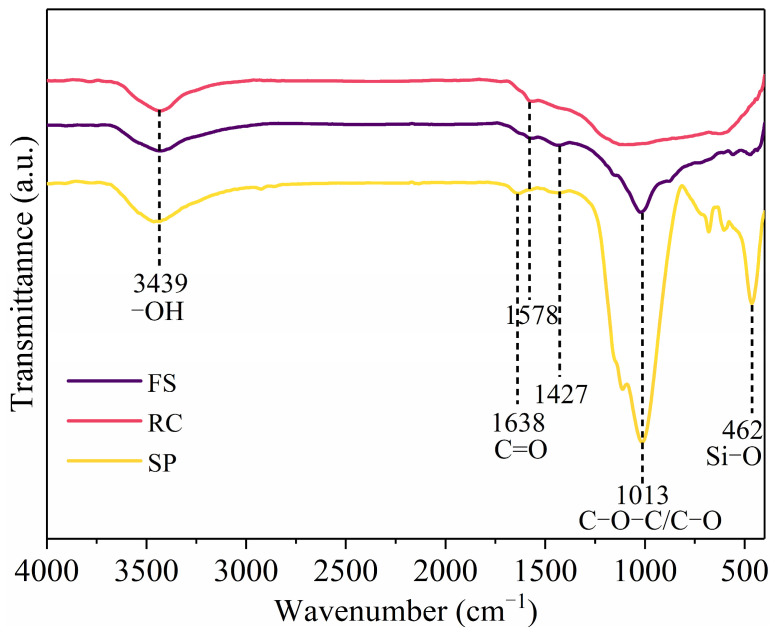
FT-IR spectra of FS, RC, and SPs.

Figure 5 provides the FT-IR spectra of the FS, RC, and SPs. The assignment of functional groups in the samples was performed by characterizing the IR spectra of the samples and comparing the absorption frequencies of the characteristic functional groups. The presence of the hydroxyl peak of free water molecules at 3439 cm^−1^ for all the samples may be due to the presence of water in the samples. The samples contained a C=O structure in the coal at 1638 cm^−1^, 1578 cm^−1^, and 1427 cm^−1^. Compared with the RC, the FS and SPs had more C-O-C/C-O structures at 1013 cm^−1^; specifically, the SP had a Si-O structure at 462 cm^−1^, which increased the hydrophilicity of the FS and SPs. The above results indicate that the high-temperature reactions in the gasifier cause significant changes to the coal surface. The presence of a large number of hydrophilic functional groups increases the difficulty in flotation decarbonization experiments [43].

**Table 5:** Summary of the typical physicochemical characteristics of FS in literature sources.

Year	Gasifier Types	Carbon Contents (%)	Ash Compositions	Mineral Compositions	Main Conclusions about Physicochemical Characteristics of FS, RC, and SPs
2024 [28]	Entrained-flow coal–water slurry gasifier	38.71	Mainly consisted of SiO_2_, Al_2_O_3_, Fe_2_O_3_, and CaO	Mainly in quartz, containing a small amount of lime	FS with a size > 180 μm showed the lowest degree of graphitization; FS in the range of 75–180 μm contained the most residual carbon; FS with a size < 45 μm exhibited the least roughness
2023 [27]	Entrained-flow coal–water slurry gasifier	25.17	Mainly consisted of SiO_2_, Al_2_O_3_, Fe_2_O_3_, and CaO	Mainly in anhydrite, quartz, and hematite	RC in FS was mainly distributed in the semimolten flocs; FS contained a lot of Si-O bonds and Al-O bonds, leading to a negative charge on the mineral surface
2022 [38]	Opposed multi-burner coal–water slurry gasifier	16.83	Mainly consisted of SiO_2_, Al_2_O_3_, Fe_2_O_3_, and CaO	Mainly in quartz and nepheline	FS with a size > 115 μm and in the range of 38–75 μm contained the most residual carbon with a higher degree of disorder; FS in the range of 75–115 μm and 0–38 μm mainly contained small-sized spherical mineral particles, which tended to adhere to the pore interior of the large-sized RC
2022 [16]	Four-nozzle coal–water slurry gasifier	18.74	Mainly consisted of SiO_2_, Al_2_O_3_, Fe_2_O_3_, and CaO	Mainly in amorphous phase and a minor proportion of crystal phase (mainly quartz)	Distribution modes of RC–ash: discrete distribution, embedded distribution, crosslinked distribution, and association and bonding; RC contained a hierarchical microporous/mesoporous/macroporous structure
2022 [44]	Entrained-flow coal–water slurry gasifier	29.50	Mainly consisted of SiO_2_, Al_2_O_3_, Fe_2_O_3_, and CaO	Amorphous aluminosilicate 96.8%, quartz 1.4%, calcite 1.8%	RC content increased with the increase in the particle size; Many inorganic spherical particles adhered to the surface and inner pores of RC; A few carbons had melted and were wrapped inside the inorganic microspheres
2021 [45]	Entrained-flow coal–water slurry gasifier	17.7	Mainly consisted of SiO_2_, Al_2_O_3_, and CaO	Mainly in glass and amorphous phase, containing quartz crystals	FS appeared to be small fragmented spheres; Many heavy metals were more concentrated in FS
2020 [15]	Entrained-flow pulverized coal gasifier	17.8	Mainly consisted of SiO_2_ and Al_2_O_3_ followed by Fe_2_O_3_ and CaO, which were approximately 7% and 8%, respectively	Mainly in amorphous aluminosilicate and a small amount of quartz	FS contained mostly amorphous aluminosilicate, together with a small amount of crystal quartz; RC contained a low degree of crystalline order; RC content increased with a particle size between 23 and 120 mm.
2020 [46]	Entrained-flow coal–water slurry gasifier	30.0	Mainly consisted of SiO_2_, Al_2_O_3_, Fe_2_O_3_, and CaO	Mainly in quartz, augite, and feldspar	The composition of FS with different size ranges showed significant differences; A significant difference in the number and size of glassy particles between the high-carbon and high-ash fractions

#### 2.1.8. In Situ Morphology Analysis

**Figure 6:**
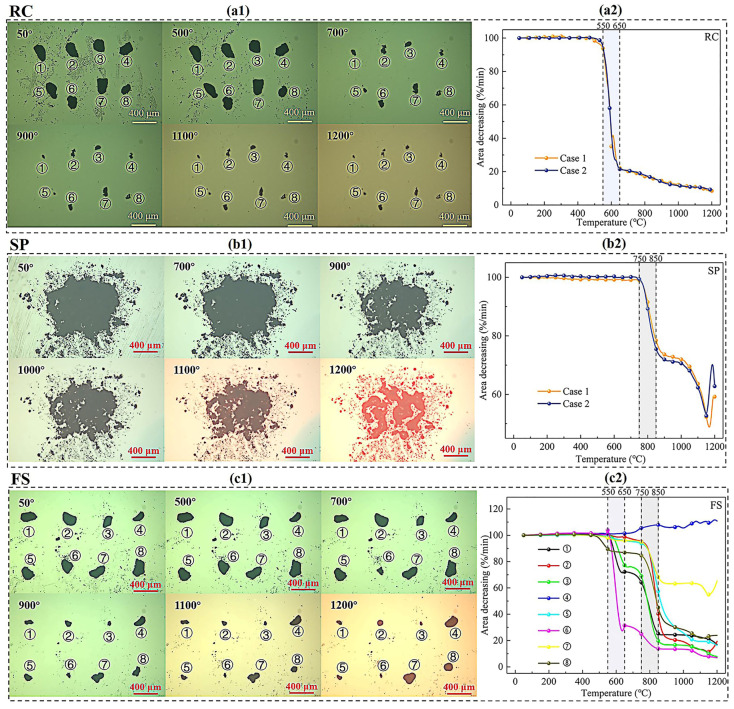
The morphology changes and decrease in area of RC, SPs and FS on 100–200 mesh. (The (**a1**,**b1**,**c1**) represent the morphology changes of RC, SPs, and FS, respectively. The (**a2**,**b2**,**c2**) represent the area changes of RC, SPs, and FS, respectively).

A high-temperature heating stage experiment was conducted on FS, SP, and RC samples with particle sizes of 0.15–0.075 mm. Figure 6 illustrates the morphology changes and area decreases in the RC, SPs, and FS with particle sizes of 0.15–0.075 mm. For RC, the particle area began to change drastically after 500 °C and remained stable after 700 °C, as shown in Figure 6(a1). The temperature interval in which the area decreased rapidly was 550 °C–650 °C, as shown in Figure 6(a2). At 650 °C, the decrease in area was about 20%, after which the area changed less. Finally, the decrease in area dropped to less than 10%, which might be due to the mineral components beginning to melt. The above results indicate that the RC particles had basically reacted completely at 550–650 °C in the high-temperature heating stage experiment. For SP, the area began to change when the temperature exceeded 700 °C as shown in Figure 6(b1). The SP area decreased rapidly in the temperature intervals of 750–850 °C and 1000–1100 °C. At temperatures above 1100 °C, the ash began to melt, resulting in an increase in area, as shown in Figure 6(b2). For FS, the particle area began to change at 700 °C. As the temperature increased, the decrease in the area of different FS particles varied depending on the RC content, as shown in Figure 6(c1). The area of FS particles decreased faster at 550–650 °C and 750–850 °C, which is consistent with the morphology analysis of RC and SPs mentioned above, as shown in Figure 6(c2).

#### 2.1.9. Summary

Table 5 summarizes the typical physicochemical characteristics of FS from several different coal gasification processes. Due to the differences in coal types, gasification processes, and operating conditions, the physicochemical characteristics of FS in different gasifiers are significantly different. The RC mass content of all FS exceeds 15%, and some even exceed 30%. The ash compositions mainly consisted of SiO_2_, Al_2_O_3_, Fe_2_O_3_, and CaO. The main mineral phase of FS was glassy aluminosilicate mixed with quartz, mullite, and other minerals. The RC appeared as dispersed flocs in different shapes without fixed forms, while the SPs tended to form spherical particles due to the effect of surface tension. The distribution modes of RC and SPs might exist in four forms: a discrete distribution, an embedded distribution, a crosslinked distribution, and association and bonding. However, the qualitative and quantitative analyses of the distribution modes of RC and SPs in FS were limited. The reasons for the abovementioned complex distributions were not clear. Hence, in the next part of this work, in order to quantify the distribution of RC and SPs in the FS with a particular size and gasification process, an innovative calculation method is proposed to determine the mass fraction of dispersed carbon particles, dispersed mineral-rich particles, and combined carbon and ash particles.

### 2.2. The Mass Ratio of Carbon, Ash, and Carbon–Ash Combination Particles

The above results show that the reaction was completed at 650 °C for RC, while the reaction started at 750 °C for SPs. Hence, the average area of FS particles in the temperature interval of 650 °C–750 °C could be representative of the mineral component area. In this work, FS particles with an area shrinkage of more than 80% were considered to be dispersed carbon with a density of 1.94 g/cm^3^. In contrast, FS particles with an area shrinkage of less than 20% were considered to be dispersed mineral components with a density of 2.72 g/cm^3^. Particles with area shrinkage rates between 20% and 80% could be considered carbon–mineral combination materials and categorized into three levels: 20–40%, 40–60%, and 60–80%. Table 6 lists the mass ratio of carbon, ash, and the carbon–ash combination at 0.075–0.150 mm. For FS with a particle size of 0.075–0.150 mm, the mass proportions of dispersed carbon, ash particles, and the carbon–ash combination were 15.19%, 38.72%, and 46.09%, respectively. The total carbon content and density of FS particles were 39.92% and 2.38 g/cm^3^, respectively. Compared with the loss on ignition (39.42%) and density (2.40 g/cm^3^) of 0.075–0.150 mm particles in FS (Table 3), the errors were 1.27% and 0.83%, respectively, which confirms the reliability of the above calculation method. The above analysis provides basic data for determining the proportion of carbon–ash combination particles with different carbon contents in different particle sizes under initial conditions in the further separation process.

## 3. Materials and Methods

### 3.1. Material Preparation

FS was obtained from the coal–water slurry gasifier of the China National Energy Investment Group Xinjiang Chemical Co., Ltd. (Hami, China). The coal gasification process was carried out in a Texaco coal–water slurry gasifier under high-temperature conditions (about 1200–1500 °C) and high pressures. Some of the unburned carbon particles and fine mineral particles entered the black water system under the entrainment of crude synthesis gas. After a process of flash evaporation, flocculation, sedimentation, and dewatering, the FS was obtained.

**Figure 7:**
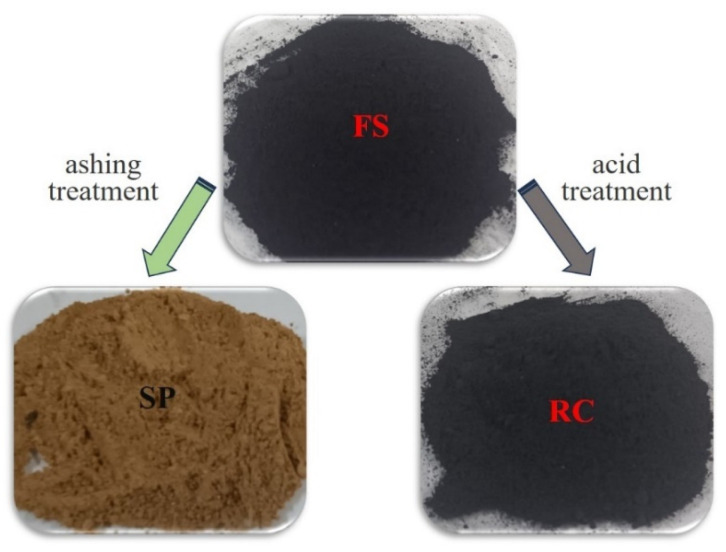
Sample schematic of FS, SPs, and RC.

Then, the FS was subjected to an acid-washing treatment and an ashing treatment, whose products are referred to as RC and slag particles (SPs), as shown in Figure 7. The acid-washing treatment process was as follows. Firstly, 80 ± 0.01 g of FS sample was treated with 36% concentrated hydrochloric acid (500 mL) for 6 h at 50 °C in a water bath with continuous agitation. Secondly, the residue was treated with 40% concentrated hydrofluoric acid (400 mL) under the same conditions. Thirdly, the residue was washed completely with distilled water until the pH = 7 and then dried at 100 °C for 12 h. The dried sample was named RC.

The ashing treatment process was as follows. Firstly, the FS sample was placed into a sealed reactor chamber of the muffle furnace. The temperature was slowly raised to 500 ± 10 °C over a period of 2 h and maintained at this temperature for 2 h. Secondly, the temperature was then further increased to 620 ± 10 °C over 2 h and held at this temperature for 2 h. The samples were stirred to expose fresh surfaces for a complete reaction and weighed every 2 h to track the loss of organic matter until the mass did not change by more than one thousandth of the mass of the residue. Finally, the residue was named SPs. The surface color of the SPs after decarburization had a reddish color, which might be due to the presence of substances, such as iron ore, in its ash composition (Table 5). In addition, to explore the relationship between unburned RC content and particle size, the FS sample was dried at 105–110 °C and then sieved into different particle size products on vibrating screening equipment.

### 3.2. Experimental Apparatus and Methods

#### 3.2.1. Loss on Burning Analysis

According to the proximate analysis of FS, the combustible material in the sample was mainly RC. Hence, the amount of residual carbon (dry basis) in the sample could be approximated by the amount of loss on combustion (dry basis). A sample with a mass of *m*_1_ was burned repeatedly in a high-temperature furnace at 820 °C until the mass was constant, which was recorded as *m*_2_. The amount of loss on burning w was calculated according to Equation (1):

(1) $$w=\frac{m_{1}-m_{2}}{m_{1}}\times 100\%$$

#### 3.2.2. Particle Size Analysis

The particle size distribution was determined by using a laser particle size analyzer (Malvern Mastersizer 2000, Malvery Instruments Ltd., Malvern, UK) with ethanol as the dispersant and a sonication time of 4 min.

#### 3.2.3. Pore Structure Analysis

The specific surface area and pore structure of the FS, RC, and SP samples were determined using a fully automatic BSD-PS(M) surface area and porosity analyzer (Beishide Instrument Technology Co., Ltd., Beijing, China), with nitrogen utilized as the adsorption gas and a degassing temperature of 150 °C maintained for 4 h. The pore size distributions of the meso and macro pores of the FS, RC, and SP samples were measured using a fully automated mercury piezometer (AutoPore V 9620, Micromeritics Instrument Corp., Norcross, GA, USA) with a measuring range of 5 nm to 800 μm.

#### 3.2.4. Scanning Electron Microscopy (SEM) Analysis

Surface morphology was analyzed using a scanning electron microscope (SEM, Tescan Mira4 LMH, Tescan Group a.s., Brno, Czech Republic) to observe the microstructure of the FS, RC, and SP samples. Due to the weak electrical conductivity of the samples, a pretreatment was required (a trace amount of the sample was stuck directly onto a conductive adhesive and sprayed with gold for 30 s using an SBC-12 sputter coater (Beijing Zhongke Keyi Co., Ltd., Beijing, China)). The morphology of the samples was then photographed at an accelerating voltage of 10.0 kV. The operating parameters were as follows: pressure of 0.00012 Pa; HV of 10.0 kV; WD of 10.2 mm.

#### 3.2.5. Mineral Phase and Functional Group Analysis

X-ray diffraction (XRD) analyses were carried out on a DX-2700B powder diffractometer (Dandong, China) using Cu Kα radiation with a scanning interval (2θ) from 5° to 80°, a scanning speed of 0.05°/min, an accelerating voltage of 40 kV, and a tube current of 40 mA. Since the samples were powders, the potassium bromide compression method was used to study the assignment of functional groups on the surface of the samples using an IR Tracer 100 Fourier Transform Infrared Spectrometer (FTIR, Vertex 70, Shimadzu Corp., Kyoto, Japan) in the range of 400–4000 cm^−1^ with a resolution of 4 cm^−1^ and 15 scans.

#### 3.2.6. In Situ Morphology Change Analysis

The morphological changes in the FS, RC, and SP samples during the heating process were recorded using a high-temperature heating stage coupled with an optical microscope system (HTSOM, DM4500P, Leica Camera AG, Wetzlar, Germany). The area changes in the sample during the heating process were calculated using the ImageJ area calculation tool. The experimental procedure was as follows. Firstly, the sapphire lens was put into the heating chamber. Then, a small number of sample particles were extracted from the sapphire lens using a tungsten needle. Finally, the particles were heated to 1200 °C in an air environment at a rate of 10 °C/min and images were recorded every minute.

#### 3.2.7. Mass Ratio of Carbon, Ash, and Carbon–Ash Combination Particles

The mass ratios of carbon, ash, and carbon–ash combination particles were calculated by the following Equations (2)–(7).

Firstly, the average area shrinkage ($\varphi_{ave}$) of a single FS particle at 650–750 °C was:

(2) $$\varphi_{ave}=\frac{\sum_{T=650}^{11}\;\varphi_{T}}{11}$$

where $\varphi_{T}$ represents the area shrinkage of particles at a temperature of *T* °C, automatically calculated every 10 °C using the ImageJ area calculation tool. Data were collected 11 times between 650 °C and 750 °C. We counted the number of particles with $\varphi_{ave}$ in the five ranges of <20%, 20–40%, 40–60%, 60–80%, and >80% and denoted them as *N_j_*.

Secondly, the mineral volume fraction of particles in the above five ranges was denoted as $V_{m,j}$:

(3) $$V_{m,j}=\frac{4}{3}\pi {\left(\frac{1-\varphi_{j}}{\pi }\right)}^{\frac{3}{2}}$$

where the area shrinkage rate of particles in each range is denoted by $\varphi_{j}$. The $\varphi_{j}$ of carbon–ash combination particles was replaced by the median area shrinkage of each range, respectively. Due to the extremely low content of volatiles and moisture in FS, the components that volatilized from the FS particles during the heating process were considered to be RC. Hence, the volume fraction of the carbon ($V_{c,j}$) in each range was as follows:

(4) $$V_{c,j}=1-V_{m,j}$$

Thirdly, the density of RC and SPs was denoted as $\rho_{c}$ and $\rho_{m}$, respectively, with values of 1.94 g/cm^3^ and 2.72 g/cm^3^. The density of the FS particles in each range could be calculated based on the proportions of the two components ($V_{m,j}$ and $V_{c,j}$), denoted as $\rho_{j}$:

(5) $$\rho_{j}=V_{c,j}\times \rho_{c}+V_{m,j}\times \rho_{m}$$

Finally, the carbon content ($\omega_{c,j}$) of each range was:

(6) $$\omega_{c,j}=\frac{V_{c,j}\times \rho_{c}}{V_{m,j}\times \rho_{m}+V_{c,j}\times \rho_{c}}$$

And the mass ratio of particles with different carbon contents in the FS was denoted as $\omega_{j}$:

(7) $$\omega_{j}=\frac{N_{j}\times \rho_{j}}{\sum_{i=1}^{5}(N_{j}\times \rho_{j})\;}$$

The density and total carbon content of the FS particles were calculated using the following Equations (8) and (9), respectively:

(8) $$\rho_{total}=\sum_{i=1}^{5}\;(\rho_{i}\frac{N_{i}}{\sum_{i=1}^{5}\;N_{i}})$$

(9) $$\omega_{total}=\sum_{i=1}^{5}\;\omega_{c,i}\omega_{i}$$

## 4. Conclusions

Clarifying the physicochemical properties and embedding forms of RC and SPs in FS is crucial for the enrichment and separation of RC in FS. This work provides a better insight into the characteristics of RC, SPs, and FS from an industrial coal–water slurry gasifier, which are important supplements to understanding the structures of FS, RC, and SPs, providing reliable technical support for effective carbon–ash separation and the subsequent comprehensive utilization of gasification slag. Our conclusions are as follows:

(1) The RC, which acted as a framework in the FS, had a rough, loose surface and a well-developed pore structure while the SPs were composed of smaller spherical particles with a smooth surface.
(2) The existence forms of RC and SPs in the FS were mainly in the form of dispersed uneven carbon, dispersed spherical SPs, agglomerated SPs, and SPs adhering within the pores of the RC. The presence of carbon–ash combination particles increased the difficulty of separation. Therefore, choosing an economical and low-energy-consuming method for its pretreatment to promote the efficient dissociation of carbon–ash combination particles is necessary;
(3) The sieving pretreatment process facilitated the enrichment of the RC in the FS. The unburned RC content showed significant dependencies according to the FS particle size. With the increase in the FS particle size, the RC content showed a trend of increasing and then decreasing;
(4) Utilizing the high-temperature heating stage coupled with an optical microscope (HTSOM) allowed for the determination of the mass percentage of dispersed carbon, dispersed ash, and carbon–ash combined particles in a specific range of particle sizes, thus providing fundamental data and reliable guidance for further separation processes.

## Figures and Tables

**Table 1:** Proximate and ultimate analysis of FS and RC (wt.%).

Sample	Proximate Analysis (wt.%)				Ultimate Analysis (wt.%)			
	M_ad_	A_d_	V_d_	FC_d_	C_d_	H_d_	N_d_	S_t,d_
FS	1.28	63.03	5.59	31.38	35.08	0.65	0.20	0.91
RC	1.75	3.22	4.50	92.28	91.68	0.71	0.57	1.02

Note: ad, air dried; d, dry basis.

**Table 2:** Ash composition analysis of FS, SPs, and RC (wt.%).

Sample	SiO_2_	Al_2_O_3_	Fe_2_O_3_	CaO	MgO	SO_3_	TiO_2_	K_2_O	ClO_2_	P_2_O_5_	Others
FS	29.51	16.33	21.71	14.1	8.06	5.16	0.86	1.33	1.00	0.79	1.18
SP	30.99	17.72	19.83	13.76	7.98	5.42	0.80	1.43	0.42	0.60	1.05
RC	1.00	5.22	3.89	21.40	13.32	42.09	0.52	0.76	10.49	0.88	0.46

**Table 3:** Yield and loss on ignition of FS at each particle fraction.

Mesh	Particle Size Range (mm)	Carbon Yield (%)	LOI (%)	Loss Ratio (%)
<35	>0.500	5.33	44.80	0.16
35–100	0.500–0.150	47.30	63.95	
100–200	0.150–0.075	20.67	39.42	
>200	<0.075	26.54	16.90	

**Table 4:** Specific surface area and pore volume of the FS, RC, and SPs.

Sample	BET Specific Surface Area (m^2^/g)	Total Pore Volume (cm^3^/g)	Microporous Volume (cm^3^/g)
FS	300.30	0.37	0.14
RC	571.52	0.61	0.29
SP	12.08	0.13	0.01

**Table 6:** Mass ratio of carbon, ash, and the carbon–ash combination at 0.075–0.150 mm.

Number	Area Shrinkage (%)	0.075–0.150 mm		
		Carbon Content (%)	Density (g/cm^3^)	Mass Ratio (%)
1	<20	3.00	2.72	38.72
2	20–40	33.54	2.40	32.42
3	40–60	56.60	2.22	6.31
4	60–80	78.39	2.07	7.36
5	>80	97.00	1.94	15.19
Total	-	39.92	2.38	100.00

## Data Availability

The data presented in this study are available upon request from the corresponding author.
